# Cell maturation influences the ability of hESC-RPE to tolerate cellular stress

**DOI:** 10.1186/s13287-022-02712-7

**Published:** 2022-01-24

**Authors:** Taina Viheriälä, Heidi Hongisto, Juhana Sorvari, Heli Skottman, Soile Nymark, Tanja Ilmarinen

**Affiliations:** 1 BioMediTech, Faculty of Medicine and Health Technology, Tampere University, Tampere, Finland; 2 Department of Ophthalmology, Institute of Clinical Medicine, University of Eastern Finland, Kuopio, Finland; 3 BioMediTech, Faculty of Medicine and Life Sciences, Tampere University, Arvo Ylpön katu 34, 33520 Tampere, Finland

**Keywords:** Human pluripotent stem cells, Retinal pigment epithelial cells, Oxidative stress, Cell therapy

## Abstract

**Background:**

Transplantation of human pluripotent stem cell-derived retinal pigment epithelium (RPE) is an urgently needed treatment for the cure of degenerative diseases of the retina. The transplanted cells must tolerate cellular stress caused by various sources such as retinal inflammation and regain their functions rapidly after the transplantation. We have previously shown the maturation level of the cultured human embryonic stem cell-derived RPE (hESC-RPE) cells to influence for example their calcium (Ca^2+^) signaling properties. Yet, no comparison of the ability of hESC-RPE at different maturity levels to tolerate cellular stress has been reported.

**Methods:**

Here, we analyzed the ability of the hESC-RPE populations with early (3 weeks) and late (12 weeks) maturation status to tolerate cellular stress caused by chemical cell stressors protease inhibitor (MG132) or hydrogen peroxide (H_2_O_2_). After the treatments, the functionality of the RPE cells was studied by transepithelial resistance, immunostainings of key RPE proteins, phagocytosis, mitochondrial membrane potential, Ca^2+^ signaling, and cytokine secretion.

**Results:**

The hESC-RPE population with late maturation status consistently showed improved tolerance to cellular stress in comparison to the population with early maturity. After the treatments, the early maturation status of hESC-RPE monolayer showed impaired barrier properties. The hESC-RPE with early maturity status also exhibited reduced phagocytic and Ca^2+^ signaling properties, especially after MG132 treatment.

**Conclusions:**

Our results suggest that due to better tolerance to cellular stress, the late maturation status of hESC-RPE population is superior compared to monolayers with early maturation status in the transplantation therapy settings.

**Supplementary Information:**

The online version contains supplementary material available at 10.1186/s13287-022-02712-7.

## Background

Retinal pigment epithelium (RPE) is a tight polarized monolayer of cells located under retinal photoreceptors at the back of the eye. RPE has many roles that altogether ensure proper visual function. The adjacent neural retina is exposed to highly oxidative environment, and due to this, one key function of RPE is to protect both itself and the retina against photo-oxidation [1, 2]. Exposure to chronic oxidative stress in the retina can lead to malfunction or death of RPE cells and retinal neurons and eventually contribute to the development of severe retinal degenerative diseases such as age-related macular degeneration (AMD) [3]. Unfortunately, current therapies mainly slow down the progression of the disease. Human pluripotent stem cell-based RPE (hPSC-RPE) transplantation is a promising approach for the treatment with safety and feasibility already under clinical investigation using either hPSC-RPE cell suspension or an intact cell sheet [4–8]. The hPSC-RPE used for cell therapy must endure high level of cellular stress caused by long time periods in cell culture, cryopreservation for cell banking, potential live-cell shipment to clinical centers, and finally transplantation-related stress including immunosuppressive drugs, inflammation, and oxidative stress in the diseased eye. Human PSC-RPE cells cultured in vitro require time to mature and to gain functions characteristic to RPE, including upregulation of genes related to antioxidant functions as the culture ages [2, 9]. Previously, the developmental stage of non-polarized cadaveric adult human RPE stem cells has been shown to affect transplantation efficacy when suspension transplantation was used, with intermediate differentiation times (4 weeks) producing the most consistent vision rescue in a rat model [10]. Polarized human embryonic stem cell (hESC)-derived RPE cells cultured for 4 weeks, on the other hand, have been shown to decrease their sensitivity to oxidative stress compared to non-polarized cells, suggesting potential advantages of sheet transplantation over the suspension approach [11]. The reported culture times for hESC-RPE sheet transplantation in clinical trials vary between 3 and 20 weeks [6]. Yet, despite potentially highly impacting therapy efficacy, the development of tolerance to cellular stress during further in vitro maturation of hESC-RPE has not been examined. Although early polarized RPE cultures may be more plastic to environmental changes than cells cultured for longer time periods, they could also be functionally more immature and not possess intact signaling pathways for critical cellular functions. Among essential RPE functions are its barrier properties that ensure the proper movement of components between the blood supply and retina [12]. With phagocytosis, RPE disposes photoreceptor outer segments (POS) that photoreceptors renew daily [13]. In addition to barrier properties and phagocytosis, intact Ca^2+^ signaling is a critical indicator of proper RPE functionality. Ca^2+^ acts as a second messenger in RPE taking part in many processes all the way from cell differentiation to cell maturation [14, 15]. Importantly, Ca^2+^ signaling is linked to purinergic signaling in RPE: extracellular adenosine triphosphate (ATP) induces elevations in intracellular Ca^2+^ concentration regulating the chemical composition and the amount of water in the subretinal space and ensuring proper communication between RPE and the retina [16, 17]. We have previously shown that culture time of hESC-RPE improves the intercellular homogeneity of Ca^2+^ response properties in cell population [12]. In the current study, we evaluated the impact of culture time on the ability of the hESC-RPE to endure treatments with chemical stressors.

The present work demonstrates how maturation level of the cultured hESC-RPE cells affects their ability to tolerate cellular stress. Under normal circumstances, one of the functions of RPE cells is to demolish the accumulation of ROS. Addition of H_2_O_2_ increases the concentration of ROS mimicking the oxidative stress environment in the cells. ROS cause oxidative damage to cellular components such as proteins which need to be removed by proteasomes. Addition of MG132 prevents the normal behavior of proteasomes leading to the accumulation of toxic protein waste. Therefore, acute oxidative stress was induced with H_2_O_2_, and as oxidative stress is also known to inactivate the proteasome in RPE [18], normal function of proteases was prevented with a protease inhibitor MG132, mimicking the effects of chronic ROS exposure. These chemical stressors were used separately. The emphasis was placed on using physiologically relevant functional assays including phagocytosis and calcium imaging to assess the properties of intact RPE monolayers maturated on porous carrier substrate for 3 or 12 weeks. Time points were chosen to represent early and late maturation status based on our previous experience and earlier study following the development of barrier function of in vitro cultured hESC-RPE over time [12]. Our results indicate that the late maturity status of hESC-RPE cells can tolerate cellular stress more effectively than the population with early maturation status, with potential implications to hPSC-RPE cell therapy.

## Methods

### Cell culture, differentiation, and treatments of hESC-RPE

Human ESC Regea08/017 (46, XX) cell line was derived, characterized, differentiated with spontaneous differentiation protocol, and cultured as previously described [19]. The cells were cultured in xeno-free (XF) conditions throughout the differentiation of hESC to RPE and the further maturation of differentiated RPE cells. XF medium, which contains KnockOut™ Dulbecco’s modified Eagle’s medium (KO-DMEM, Gibco, Thermo Fisher Scientific) supplemented with 15% xeno-free KnockOut™ serum replacement (Gibco, Thermo Fisher Scientific), 2 mM GlutaMAX™ (Gibco, Thermo Fisher Scientific), 0.1 mM 2-mercaptoethanol (Gibco, Thermo Fisher Scientific), 1% MEM non-essential amino acids (Gibco, Thermo Fisher Scientific), and 50 U/ml penicillin–streptomycin (Gibco, Thermo Fisher Scientific), were changed three times a week. Cells were cultured in 37 °C with 5% CO_2_.

For maturation, the hESC-RPE cells were thawed and seeded with a density of 2.5 × 10^5^ cells/cm^2^ on a porous polyethylene terephthalate (PET) hanging cell culture inserts (0.3 cm^2^, pore size 1.0 um, Millipore or Sarstedt) or with a density of 2.1 × 10^5^ cells/cm^2^ on a 48 well plate, depending on the experiment. The surfaces were coated with a combination of Collagen IV (10ug/cm^2^, Sigma Aldrich) and laminin 521 (1.8 ug/cm^2^, Biolamina) in phosphate saline buffer (PBS, Gibco) containing Ca^2+^ and Mg^2+^. Cells were cultured for 3 or 12 weeks. Before each experiment, the cells were exposed to 1 µm MG132 (Calbiochem) protease inhibitor or 600 µm H_2_O_2_ solution (Sigma Aldrich) diluted in medium for 24 h. Control cells obtained fresh medium without the chemical stressors.

### Mitochondrial Membrane Potential

For mitochondrial membrane potential measurements, a TMRE-Mitochondrial Membrane Potential Assay Kit (abcam, ab113852) was used according to manufacturer’s instruction. Briefly, hESC-RPE cells were thawed on a 48 well plate and cultured for 3 and 12 weeks, respectively. 600 nM tetramethylrhodamine ethyl ester (TMRE) solution diluted in culture media was used, and 20 µM carbonyl cyanide 4-(trifluoromethoxy) phenylhydrazone (FCCP) served as a negative control compound. All steps were carried under light protection. Diluted TMRE was added to the cells and incubated for 20 min at + 37 °C, and a microplate reader was used to detect the fluorescence from the TMRE. Excitation and emission wavelengths of 549/575 nm were used. Negative controls were measured for both treated and non-treated cells. Results from the microplate reader were given as intensity values and the values are presented as boxplots. Control values were set to 100% and values from the treated cells were compared to the control cells as relative change in the fluorescence intensity. Higher fluorescence intensity corresponds to a higher value of mitochondrial membrane potential. 13–16 replicate wells from 3 maturation experiments were measured at both time points.

### Transepithelial electrical resistance

Barrier development of hESC-RPE was measured from RPE cells cultured on PET hanging inserts. Transepithelial electrical resistance (TER) was measured using Millicell electrical resistance volt-ohm meter (Merck Millipore). Before each measurement, the cells were equilibrated for 10 min at room temperature (RT). Each hESC-RPE monolayer was measured twice, and the average TER values were calculated. PET insert without cells was used as a background and was subtracted from each TER value. Measurements were obtained at time points of 3 weeks and 12 weeks. In each insert, relative change was obtained by subtracting the value before the treatment from the value after the treatment and dividing this difference by the value before the treatment. Measurements were obtained from the control cells similarly and changes in these values were subtracted from the values of treated cells to obtain the final relative change caused by the treatments and compared to the controls. 6–14 replicate inserts from 2 to 3 maturation experiments were measured at both time points.

### Immunofluorescence stainings

The hESC-RPE monolayers cultured on inserts were fixed for immunostainings at time points of 3 and 12 weeks. All steps were conducted at RT unless otherwise stated. The fixation was performed with 4% paraformaldehyde (Sigma Aldrich) in PBS for 15 min after which the cells were permeabilized with 0.1% Triton X-100 (Sigma Aldrich) in PBS for 15 min. This was followed by blocking with 3% bovine serum albumin (BSA, Sigma Aldrich) in PBS for 1 h. Primary antibodies for Zonula occludens (ZO-1, 1:200, 61-7300, Invitrogen), Na^+^/K^+^-ATPase (1:200, ab7671, Abcam), Connexin 43 (Cx43, 1:200, ab11370, Abcam), P2Y_2_ (1:200, PA1-46150, Invitrogen) and claudin-19 (CL19, 1:200, MAB6970, R&D) were diluted in 3% BSA in PBS and incubated overnight at + 4 °C. The samples were washed several times with PBS followed by 1 h incubation with secondary antibodies donkey anti-mouse Alexa Fluor 488 (1:200, A21202, Life Technologies) and donkey anti-rabbit Alexa Fluor 568 (1:200, A10042, Life Technologies). F-actin was stained with Phalloidin (1:800, P1951, Sigma Aldrich). After incubation, the samples were washed with PBS and mounted with ProLong™ Gold Antifade Mountant with DAPI (Invitrogen, Thermo Fisher Scientific).

Z-stack images from the immunofluorescence-stained samples were captured with laser scanning confocal microscopes Zeiss LSM700, LSM780, or LSM800 with 63x/1.4 oil immersion objective. Images were converted to maximum intensity projections (MIP) with ImageJ [20, 21]. 2–5 replicate inserts from 2 to 3 biological replicates were immunostained and imaged at both time points.

### Phagocytosis assay

POS fragments were isolated and purified from porcine eyes as described in [19]. The fragments were suspended in RPE medium containing 10% fetal bovine serum (FBS, Gibco). The POS-media were then added to the apical side of the cells cultured on inserts and incubated for 2 h at 37 °C with 5% CO_2_. After this, the cells were washed with PBS, fixed, and immunostained as described above. POS particles were immunolabelled with primary antibody anti-opsin (1:1000, O4886, Sigma Aldrich) and actin filaments were stained with Phalloidin (1:800). 2–3 replicate inserts from 2–3 maturation experiments were used at both time points.

Z-stack images were acquired with laser scanning confocal microscope Zeiss LSM800 with 63x/1.4 oil immersion objective with interval of 100 nm. From each sample, five Z-stack images were captured from randomly selected areas. These images were resliced with ImageJ to 512 xz-slices which were converted to MIPs of 20 consecutive xz-slices, from which the internalized POS particles were then manually calculated. In addition, the overall number of cells was calculated to obtain the average number of POS particles per cell.

### Ca^2+^ imaging

For Ca^2+^ imaging, hESC-RPE cells were cultured on hanging inserts. Ca^2+^ imaging of the hESC-RPE monolayers was performed as previously described [12, 22] at time points of 3 weeks and 12 weeks. Briefly, the cells were loaded with Ca^2+^ sensitive dye fluo-4-acetoxymethyl ester (1 mM, fluo-4 AM; Molecular Probes, Thermo Fischer Scientific) for 45 min. Elliot buffer solution (pH 7.4, 330 mOsm) was used for washing the cells. During the imaging, the cells were perfused with Elliot alone or Elliot containing 100 µM ATP (Sigma Aldrich) with gravity-fed solution exchange system (AutoMate Scientific). All steps were performed at RT protected from light. Nikon Eclipse FN1 upright fluorescence microscope with a 25 × water immersion objective (NA = 1,10) was used for imaging. The cells were imaged for total of 10 min which included 2 min of baseline, 2 min of ATP stimulus, and 6 min of additional imaging. Data analysis wasconducted from three randomly selected regions of interest (ROIs). Each ROI was 200 × 200 pixels (104 × 140 µm) and from each ROI (approx. 200 cells/ROI) cells were outlined in ImageJ [20, 21]. The intensity data as a function of time was converted to a MATLAB (R2018b) form and analyzed using a script package as in [22]. Each cell was categorized in one of the two groups: cells that respond by Ca^2+^ elevation to the ATP stimulus and cells that do not respond to it. Responding cells were then further analyzed to determine the relative maximum amplitude of the Ca^2+^ response. 2–5 replicate inserts from 2 to 3 maturation experiments were measured at both time points. In each experiment day, both control inserts and inserts after the treatments were measured to overcome the possible effect of slight differences in the flow rate between experiment days visible in the data as a latency difference (see Fig. 5).

### Cytokine array kit

Cells were cultured on hanging inserts, and at both time point, apical culture media were collected and frozen at – 80 °C. Secretion of multiple cytokines was measured from the collected media with Cytokine array kit (ARY005B, R&D Systems). 350 µl of media were used per sample and the assay was performed following manufacturer’s protocol. Membranes were imaged with Bio-Rad X ChemiDoc XRS + with exposure time of 5–11 min (exposure time in which the control spots had reached maximal grey value were chosen). Intensities of the spots within each experiment were compared by using ImageJ and the results are presented as bar graphs. 2–4 replicate media samples from 2 biological experiments were measured at both time points. Only cytokines whose change compared to the control were congruent between 2 replicates were included.

### Statistical analysis

All statistics were performed with Mann–Whitney *U* with GraphPad Prism (version 5.02) test to compare statistical significances. A *p*-value of < 0.05 was considered statistically significant.

## Results

### Mitochondria remained active after treatments with MG132 and H_2_O_2_

**Fig. 1:**
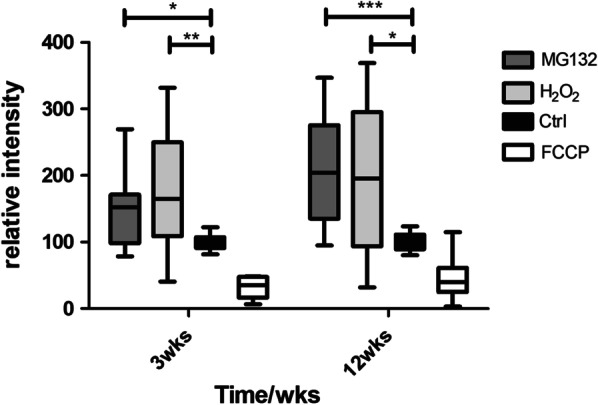
Relative intensity of mitochondrial membrane potential values at time points of 3 weeks and 12 weeks. MG132 and H_2_O_2_ treated cells were compared to the control cells. FCCP represents a negative control compound. Mean of the control was set as 100%. Data represents means ± SD values from 13 to 16 replicates. *p < 0.05, **p < 0.01 and ***p < 0.001

To assess functional consequences of the stressors rather than cell death, hESC-RPE cells cultured for 3 weeks (early maturation status, TER > 160 ohmcm^2^) or 12 weeks (late maturation status, TER > 840 ohm cm^2^) were treated with sublethal concentrations of MG132 or H_2_O_2_, as previously determined for mature hESC-RPE [23, 24]. Mitochondria are important organelles for the cell’s normal behavior and highly susceptible to oxidative damage. It is known that mitochondrial membrane potential decreases in apoptosis [25]. To evaluate cell viability and to analyze the mitochondrial activity, mitochondrial membrane potentials were measured. TMRE accumulates to active mitochondria and can be visualized as increased fluorescence intensity. The measured intensities between the control and cells treated with MG132 or H_2_O_2_ differed significantly at both time points (MG132 at 3 weeks *p* = 0.0186 and 12 weeks p < 0.0001, H_2_O_2_ 3 weeks *p* = 0.0039 and 12 weeks *p* = 0.0210). Cells after both treatments and at both time points revealed active mitochondria, on average with increased membrane potential compared to the control, indicating sublethal treatment range for both 3- and 12-week cultures (Fig. 1). Interestingly, there was larger variation in the values in the treated cells than in the control cells.

### hESC-RPE with early maturation status is more susceptible to stress-induced changes in cellular barriers

**Fig. 2:**
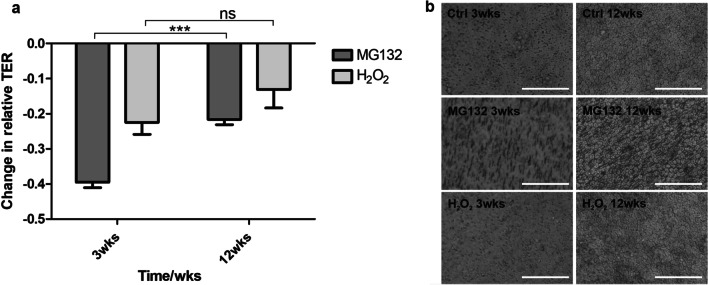
**a** Relative change in TER values induced by the treatment with the stressors for early (3 weeks) and late (12 weeks) maturation status of hESC-RPE. Data shows the relative change in TER values before and after the treatments with MG132 and H_2_O_2_. Data represents means ± SD values from 6 to 14 replicates. Values that differ statistically are shown in the image with asterisks. **p < 0.01 and ***p < 0.001. **b** Phase contrast images of MG132 and H_2_O_2_ treatments and untreated hESC-RPE monolayers at time points of 3 weeks and 12 weeks. Images were taken from live cultures in inserts immediately after the treatments. At 3-weeks, the pores from the culture inserts are visible since the pigmentation of hESC-RPE cells is relatively low. At 12-weeks, hESC-RPE cells have gained pigmentation and therefore the pores are not visible. In addition, the cobblestone morphology of the hESC-RPE cells is clearer at 12-weeks compared to 3-weeks. Scale bar is 100 µm

The effects of MG132 and H_2_O_2_ on hESC-RPE’s barrier properties were assessed by TER measurement and immunostainings with anti-ZO-1 and anti-CL19 antibodies. The stressed cells showed lower TER values compared to control cells at both time points (Fig. 2). However, the impact of the treatments on the tight junctions was larger with the cells of early maturation status. The MG132 treated cells had 45% ± 9 lower TER values than control cells (*p* = 0.0001) at the time point of 3 weeks. At 12 weeks the MG132 treated cells had only 22% ± 5 lower TER values than the control cells (*p* = 0.0002). Similarly, the treatment with H_2_O_2_ showed significantly lower TER values compared to the control cells at 3 weeks (22% ± 8 decrease, *p* = 0.0056) but at 12 weeks the decrease was not statistically significant (12% ± 14, *p* = 0.0825).

**Fig. 3:**
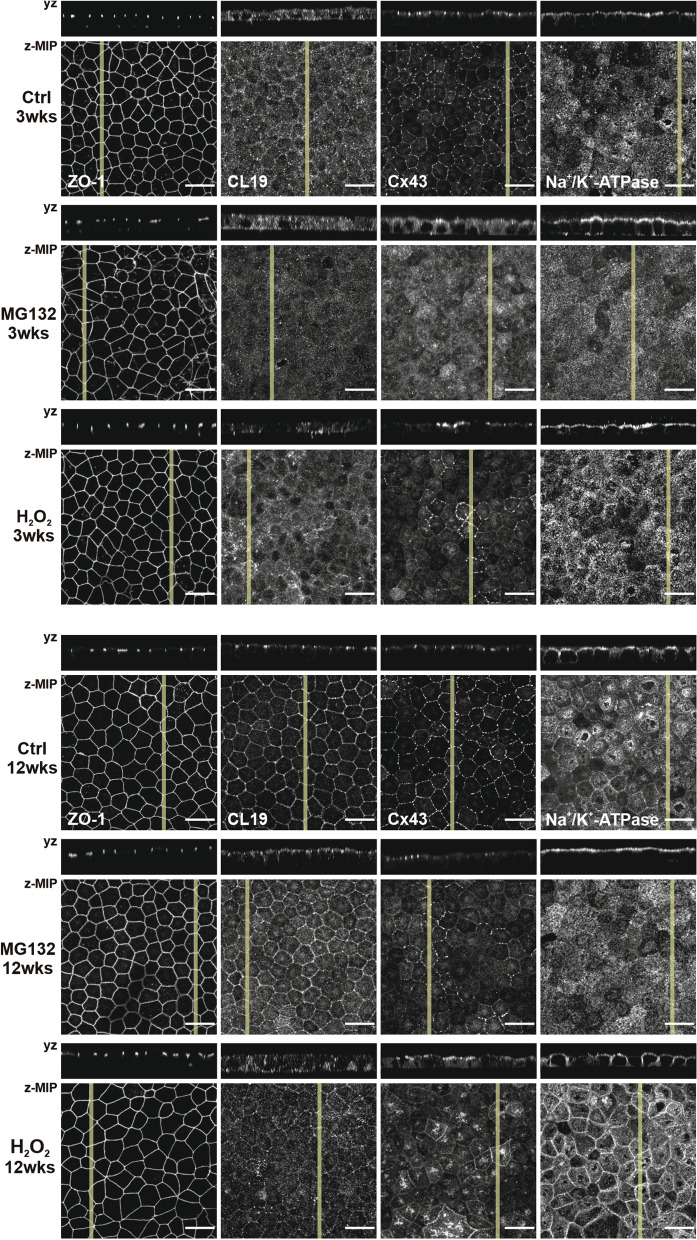
Immunofluorescence images of localization of RPE proteins in control, MG132 and H_2_O_2_ treated hESC-RPE cells at time points of 3 weeks and 12 weeks. Each image consists of a laser scanning confocal microscopy *z*-maximum intensity projection (*z*-MIP) and *yz* cross-sections (MIP from 10 sections). Scale bar is 20 µm

Of the tight junction proteins, ZO-1 is associated with the junctions from the early maturation process on, and its cellular localization was not affected by either of the treatments. Claudins, on the other hand, especially CL19 in RPE cells, are involved in the determination of the junctional properties, such as permeability and selectivity [26]. In early maturation status of hESC-RPE cells, CL19 is located on the apical surface whereas in polarized cells the localization is shifted to the tight junctions (as seen in control cells in Fig. 3). Consistent with the TER values, hESC-RPE cells treated with both MG132 and H_2_O_2_ showed a shift in the localization of CL19 from the junctions to the cytoplasm with the highest impact seen at 3 weeks.

Among interepithelial junctions are gap junctions that regulate cell–cell communication. Cx43 is a major gap junction protein expressed by RPE, and it has been reported that protection of RPE cells from oxidative stress-induced death is dependent on functional Cx43 channels [27]. In late maturation status, polarized RPE, Cx43 can be visualized as punctuate structures at the cell borders. MG132 treatment shifted the localization of Cx43 from the junctions to the cell membranes at 3 weeks, whereas at 12 weeks, the localization remained punctuate in junctions, yet, being not detectable in some cells. After the H_2_O_2_ treatment, the Cx43 localization was quite heterogenous, especially at the time point of 3 weeks. In some cells, Cx43 could not be detected indicating loss of expression, and in some cells the localization was shifted to apical membrane or the cytoplasm. At 12 weeks, Cx43 was accumulated in the apical surface.

In addition to the maintenance of transepithelial gradient, Na^+^/K^+^-ATPase has been shown to play a role in the function of tight junctions in mammalian cells, including RPE [28–30]. Similarly to native RPE, Na^+^/K^+^-ATPase is normally apically polarized in hESC-RPE. Na^+^/K^+^-ATPase localization after MG132 treatment shifted more visibly to the apical membrane at both time points. Though, 3-week time point revealed also basolateral localization. H_2_O_2_ treatment did not have a major impact on the expression nor on the localization of Na^+^/K^+^-ATPase. Despite the changes in the TER, especially after MG132 treatment, and expression and localization of specific proteins, notable changes in the morphology and pigmentation were not observed (Fig. 2b). Morphological changes were further confirmed with confocal imaging (Fig. 3).

### hESC-RPE with early maturation status are susceptible to MG132 but not H_2_O_2_-mediated reduction in phagocytosis activity

**Fig. 4:**
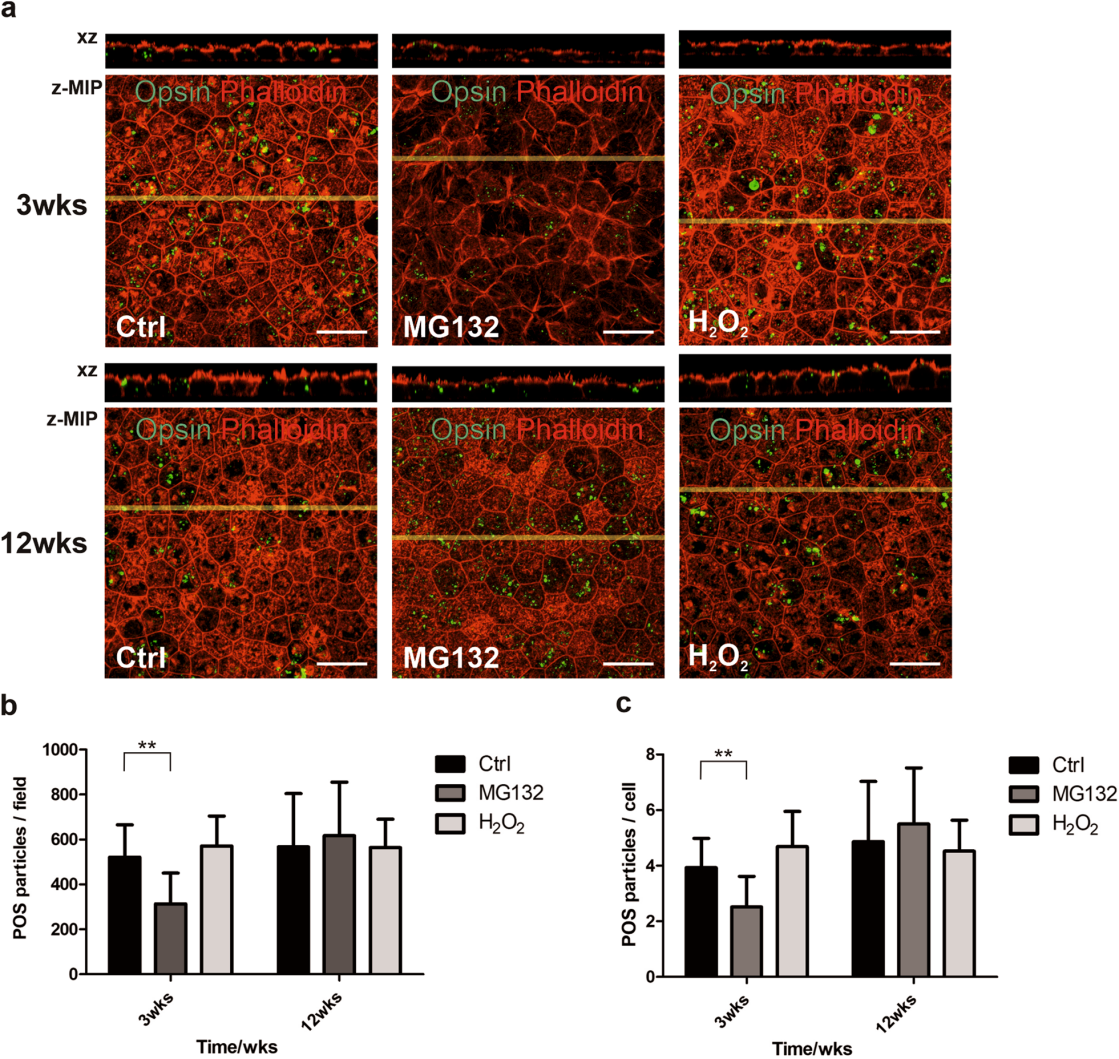
Phagocytosis assay of hESC-RPE at time points of 3 weeks and 12 weeks. **a** Each image consists of a laser scanning confocal microscopy *z*-maximum intensity projection (*z*-MIP) and *xz* cross-sections (MIP from 20 sections) with POS particles (opsin staining) shown in green and F-actin (phalloidin staining) shown in red. Scale bar is 20 µm. POS particles inside the cells were calculated from xz cross-sections and are shown as **b** number of internalized POS particles per field (total field number 10–15 from 2 to 3 experiments) and **c** number of internalized POS particles per cell. Each field contains ca. 100 cells. Internalized POS particles were calculated as in [12]. Data represent means ± SD. **p < 0.01

Previously, sublethal oxidative stress has been reported to reduce phagocytosis in an immortal RPE cell line ARPE19 [31]. Yet, the effect of oxidative stress to phagocytosis of hPSC-RPE cells is unknown. The susceptibility of hESC-RPE phagocytosis efficiency to cellular stress at different maturation stages was examined by exposing the cells to isolated porcine POS for 2 h immediately after 24-h MG132 or H_2_O_2_ treatments. The number of internalized POS particles identified by anti-opsin was counted from xz confocal images. Compared to control cells, only the MG132 treatment affected the POS intake efficiency and only in the 3-week cultured cells, indicative of a rather robust nature of the hESC-RPE phagocytic machinery, especially at the more advanced maturation stage (Fig. 4).

### Ca^2+^ signaling was reduced especially in early maturation status after MG132 treatment

**Fig. 5:**
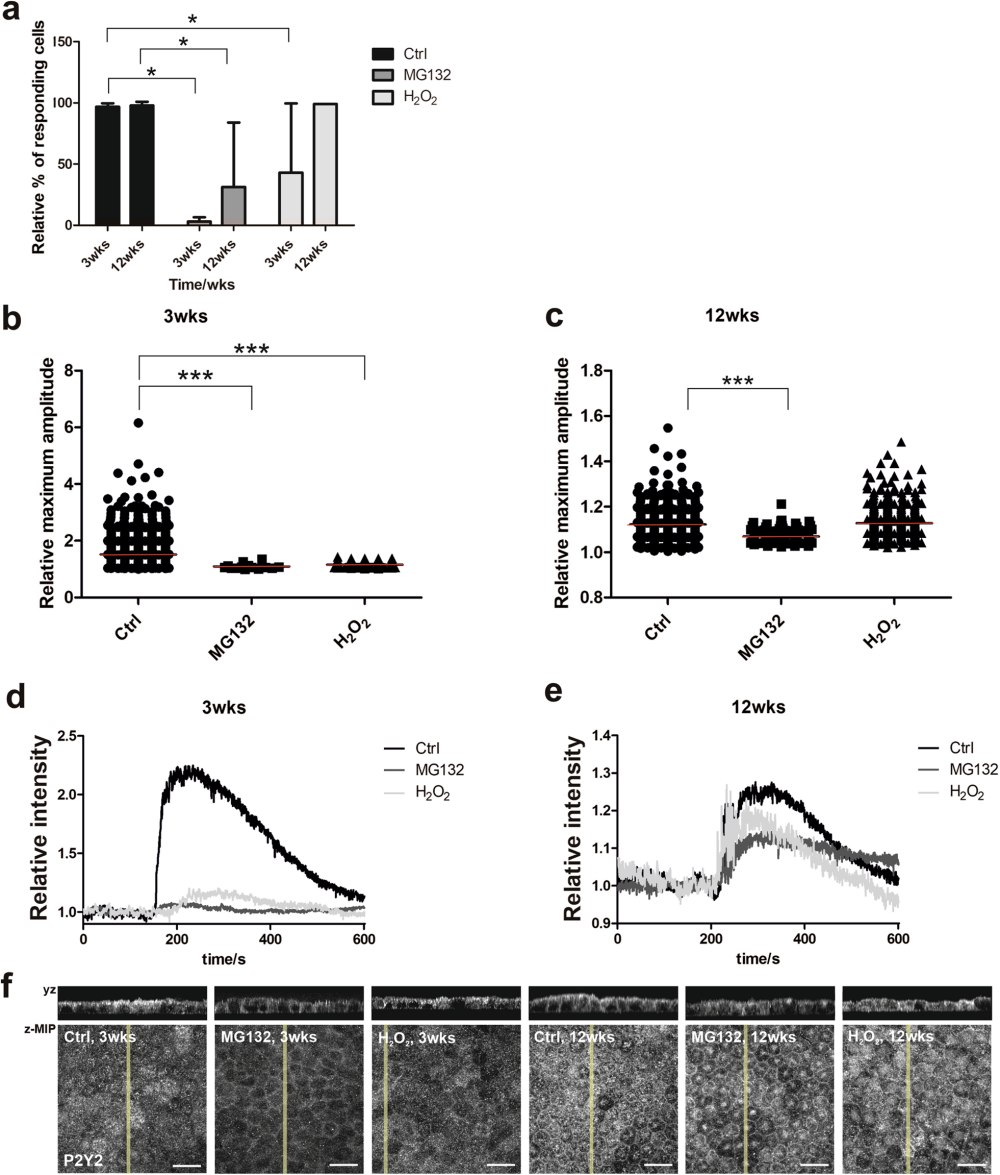
Ca^2+^ signaling of control, MG132 and H_2_O_2_ treated hESC-RPE cells at time points of 3 and 12 weeks. Images represent **a** relative number of cells that respond to ATP stimulus at both timepoints, and relative maximum amplitudes at timepoints of **b** 3 weeks and **c** 12 weeks. Representative response curves at the timepoints of **d** 3 weeks and **e** 12 weeks. All results are conducted from 2 to 5 measurements. All data represents means ± SD. *p < 0.05 and ***p < 0.001. **f** Representative laser scanning confocal microscopy *z*-maximum intensity projections (*z*-MIP) and *yz* cross sections (MIP from 10 sections) of expression and localization of P2Y_2_ receptor. Scale bar is 20 µm

We and others have previously shown that examining Ca^2+^ signaling properties is a sensitive method for evaluating the quality and functionality of hPSC-RPE [12, 32]. In this study, Ca^2+^ signaling was analyzed by counting the number of cells responding to ATP stimulus by intracellular Ca^2+^ transients (Additional file 1: Fig.S1a and S1b) and calculating the relative amount of the responding cells (Fig. 5a). In addition, maximum response amplitudes as intensities relative to the baseline were analyzed. In control cells, ATP-induced Ca^2+^-responses were extensively detected at both time points (3 weeks: 97% ± 3, 12 weeks: 98% ± 3), although a large variation in the maximum amplitudes was observed at the earlier time point (Fig. 5b) indicating heterogeneity of cells in the monolayer. At 12 weeks’ time point, control cells were more homogenous regarding the maximum amplitudes (Fig. 5c). At 3 weeks, merely a few percent of cells treated with MG132 responded to the ATP stimulus by Ca^2+^ transients (3% ± 3) while at 12 weeks, this number was tenfold higher (31% ± 43). Cells treated with H_2_O_2_ showed overall more intracellular Ca^2+^-activity compared to the MG132 treated cells: At 3 weeks, 43% ± 33 responded to the ATP stimulus and at 12 weeks, practically all cells (99% ± 0) responded to the ATP stimulus. Representative response curves from both time points are illustrated in Fig. 5d, e.

In addition to changes in the percentage of responding cells, the treatments induced changes in the response amplitude as well. At 3 weeks (Fig. 5b), both MG132 and H_2_O_2_ resulted in significantly lower maximum amplitudes compared to the control cells (*p* < 0.0001). At 12 weeks (Fig. 5c), MG132 treated cells remained in significantly lower levels in their Ca^2+^ response amplitudes compared to the control cells (*p* < 0.0001), but the cells treated with H_2_O_2_ showed no significant difference to the controls.

Addition of ATP to the apical side of RPE induces an intracellular Ca^2+^ transient primarily via apical P2Y_2_ receptors [16]. In control cells, P2Y_2_ was detected at the apical and lateral membrane at both timepoints (Fig. 5f). Interestingly, both treatments influenced the cellular localization of these receptors. At 3 weeks, the treatments shifted the localization of P2Y_2_ away from the apical membrane towards the cytoplasm and the cell–cell junctions, especially with the MG132 treatment. At 12 weeks, the transition from the apical localization to the cytoplasmic was even further enhanced. In addition, the staining appeared homogenous around the monolayer in the control hESC-RPE, whereas the treated cells, especially with H_2_O_2_, showed heterogenous staining pattern containing cells in the monolayer with no P2Y_2_ protein expression.

### Cytokine expression during hESC-RPE maturation and stress induction

**Fig. 6:**
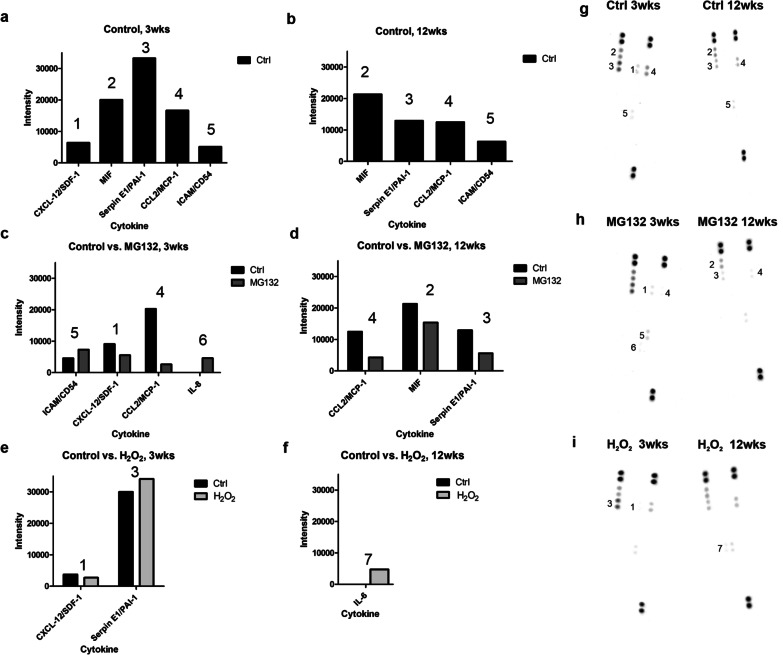
Cytokine secretion of hESC-RPE cells. Intensity levels of secreted cytokines of control cells at **a** 3 weeks and **b** 12 weeks. Control values are values from membranes within each experiment (*n* = 2 for 3 weeks and *n* = 1 for 12 weeks). Intensity levels after MG132 treatment at **c** 3 weeks and **d** 12 weeks and after H_2_O_2_ treatment at **e** 3 weeks and **f** 12 weeks. **g**–**i** Cytokine array membranes illustrating spot intensities. Replicate experiment is shown in Additional file 2: Fig. S2

Paracrine signaling is an important way for the cells to communicate in different situations like inflammation. Especially in the cases where the ocular immune privilege has been disrupted either due to disease or surgical intervention, the expression of inflammatory cytokines or chemokines by the transplanted cells could increase the possibility of graft rejection. In our study, the untreated cells secreted a variety of different cytokines at both time points (Fig. 6a, b, Additional file 2: Fig. S2a and S2b, Additional file 3: Fig. S3a and S3b). Majority of the cytokines expressed at 3 weeks were downregulated at 12 weeks compared to the 3-week time point. Cell population with late maturation status revealed downregulated secretion of macrophage migration factor (MIF), plasminogen activator inhibitor-1 (Serpin E1/PAI-1) and monocyte chemoattractant protein-1 (CCL2/MCP-1) compared to cells with early maturation status. Stromal cell-derived factor 1 (CXCL12/SDF-1) was secreted only at the time point of 3 weeks. Intracellular adhesion molecule 1 (ICAM/CD54) was slightly upregulated at 12 weeks compared to the 3-week time point.

At the same time, the secretion levels of these cytokines were modestly altered after the treatment with MG132 or H_2_O_2_ (Fig. 6c–f, Additional file 2: Fig. S2c–f). Notably, expression of IL6 and IL8 were revealed only after the treatments. IL6 expression was observed repeatedly at the 12-week time point after H_2_O_2_ treatment (Fig. 6f, Additional file 2: Fig. S2f) and IL8 at the 3-week time point after MG132 treatment.

## Discussion

Transplantation of RPE cells is a potential treatment strategy for retinal diseases such as AMD. The ability to differentiate RPE from hPSCs has offered a renewable cell source for therapeutical applications. For effective treatment, hPSC-RPE must exhibit the physiological characteristics of native human RPE. For therapeutic efficacy, it is important that the hPSC-RPE cells remain viable and are functional rapidly after transplantation in the challenging subretinal environment and in the presence of several additional stressors e.g. from the transplantation procedure itself and the environment of the diseased retina. Previously, hESC-RPE cell maturation level has been shown to affect their sensitivity to oxidative stress-induced cell death, however, the maturation status was evaluated only based on TER [11]. In addition, maturation level has been shown to affect transplant efficacy, but this has been evaluated only using the suspension transplantation approach [10]. In this study, most of the analyses were conducted with PET carriers which allow RPE maturation in monolayer format with physiologically relevant nutrient flow from the basolateral side and has also been used in transplantation setting [33]. We aimed to analyze the consequences of sublethal cellular stress induced by H_2_O_2_ and proteasome inhibitor MG132 to hESC-RPE with early versus late maturation status. Based on our previous experience with hESC-RPE, expression and junctional localization of CL19 along with TER measurements are good indicators of functional maturation of the cells in culture, proper barrier function being a crucial property of RPE. Thus, in the current study, the maturation level was chosen mainly using these criteria and in accordance with our previous observations, junctional localization of CL19 was observed only after the 12-week culture time. Exposure to H_2_O_2_ is a widely used method to cause oxidative stress in cellular models and in RPE cells [34]. The ubiquitin-proteasome system on the other hand is responsible for degradation of damaged or redundant proteins which would otherwise accumulate as cellular debris and can be inactivated by oxidative stress [35].

Mitochondrial damage is adequate to initiate the degeneration of RPE leading to diseases such as AMD [36]. Mitochondria react rapidly and reversibly to many triggers from inside and outside the cell and have a key role for example in the activation of cell death [25]. Hence, we initiated this work by measuring the mitochondrial membrane potential after the treatment with MG132 or H_2_O_2_ to evaluate the fatality of the treatments, since mitochondrial membrane potential should decrease or cease in cell death [25]. Neither treatment showed signs of membrane potential decrease indicating that the treatments were not fatal to the cells. However, mitochondria protect themselves and other cellular components from oxygen damage [34] by activating defense mechanisms in response to cellular stressors. The subsequent increase in the need for energy could explain the increase in the mitochondrial membrane potential observed after the treatments. However, as cell populations are heterogenous in their responses, variation in the treated cells was large and included cells with remarkably lower membrane potentials compared to control.

RPE acts as a barrier between the choroid and subretinal space which is needed for proper neural homeostasis [37]. Tight junctional complexes between neighboring RPE cells ensure the proper functioning of this barrier. Tight junctions form in the apical periphery of contacting cells mediating the diffusion from the choroid to the subretinal space and vice versa. ZO1 and especially CL19 are responsible for the formation of the tight junction barrier in RPE and thereby take part in the modulation of transepithelial diffusion [38]. Disruption of this barrier enables an uncontrollable leakage of molecules and nutrients [37]. Transepithelial barrier can be measured with TER that we have previously shown to slowly increase during maturation as the tight junctions mature [12]. In addition, induction of oxidative stress has been shown to reduce TER which is usually a result of cell death [11, 24, 39]. In this study, as the TER reduced dramatically at 3 weeks after MG132 treatment, but the mitochondrial activity did not indicate cell death, tight junctions were analyzed in more detail. CL19 subcellular localization was altered at both time points with both chemical treatments, especially at 3 weeks after MG132 treatment. While CL19 localization was altered, the localization of ZO1 remained intact. Previously, Liu et al. [26] have demonstrated that CL19 knockdown has a similar effect with reduced TER and ZO1 remaining associated to apical junctional complex. Knockdown of CL19 has also been shown to affect phagocytosis [26], and similarly, in our study, the most pronounced reductions in CL19 junctional localization and in phagocytosis activity were correlated. Knockdown of CL19 reduced the rate of degradation but not binding or ingestion of POS in hiPSC-RPE [26]. However, in our study, the phagocytosis rate was analyzed after a short, 2-h incubation with POS, suggesting that the detected reduction in phagocytosis is more likely to reflect earlier events such as POS binding and/or internalization than degradation. Malfunctions in phagocytosis can lead to accumulation of POS particles and lipofuscin which together generate an enormous amount of reactive oxygen species (ROS) [26, 40]. One cause for the initiation of AMD is suggested to be the inability of RPE cells to demolish ROS [24]. Interestingly, in the study by Liu et al. [26], knockdown of CL19 activated AMPK, a protein kinase known to be activated by oxidative stress and linked to MER tyrosine kinase (MERTK) inactivation and inhibition of the internalization of POS in ARPE19 cells [41]. In the current study, phagocytosis was only reduced at 3-week time point and only with the MG132 treated cells. MG132 has been shown to activate AMPK in several cell types which were diminished by antioxidants [42], potentially suggesting a more developed antioxidant defense system in the more mature hESC-RPE at the 12-week time point.

In addition to tight and adherent junctions, cell–cell contacts in RPE consist of gap junctions. The gap junctions are formed by hemichannels, the basic components of which are connexins, Cx43 being the most widely expressed [27]. In the control hESC-RPE, Cx43 was localized in a similar manner on the apical and intercellular membrane than previously reported for mouse RPE and ARPE19 cells [43]. Cx43 has been suggested to have a protective role against oxidative stress, also in RPE cells where Cx43 knockdown increased the susceptibility of ARPE19 cells to oxidative stress-induced cell death [27, 44]. Oxidative stress was reported to alter the expression and subcellular localization of Cx43 in RPE by increasing its cytoplasmic aggregation, as was also seen in our study, especially in the cells with early maturation status followed by MG132 treatment and cells with late maturation status after H_2_O_2_ treatment. Connexins have a half-life of only a few hours and depending on their stage of assembly, connexins can be degraded through different pathways [45]. In many cell types, treatment with proteasomal inhibitors leads to an increase in Cx43 immunoreactivity aggregates suggesting a role for proteasomes in the degradation of gap junctions. Gap junctions are also degraded via autophagy, an important homeostatic mechanism shown to be functional in hESC-RPE [23]. In many cell types, including RPE, proteasome inhibition seems to upregulate autophagy [46]. Although the maturation rate of the autophagic machinery in hESC-RPE during differentiation is not yet known, immature autophagy at the 3-week time point could at least partly explain the accumulation of Cx43 in the cells with early maturation status after MG132 treatment compared to the more mature hESC-RPE.

Na^+^/K^+^-ATPase maintains the transepithelial gradient [28]. In this study, the expression and apical localization of Na^+^/K^+^-ATPase were increased after the MG132 treatment. Proteins localized to plasma membranes are degraded via endocytosis but proteasomal inhibitors are known to inhibit endocytosis as well as degradation of proteins [47, 48]. The halftime of plasma membrane Na^+^/K^+^-ATPase in alveolar epithelium is 4 h [47]. Assuming that the regulation of Na^+^/K^+^-ATPase life cycle in RPE is similar, accumulation of Na^+^/K^+^-ATPase in the apical membrane within the timeframe of 24-h MG132 incubation can be perceived due to the malfunctions in endocytosis.

Purinergic signaling is important for the integrative functions of the retina and RPE. Intact ATP signaling can be considered as one key indicator of the hPSC-RPE authenticity, a prerequisite for the successful outcome of RPE transplantation therapy [32]. With our previously developed analysis tools for Ca^2+^ imaging [22], control cells demonstrated the ability to respond to the ATP stimulus already at the earlier time point. Further analysis revealed that the Ca^2+^ responses of the hESC-RPE with late maturation status cells were less exposed to the treatments compared to the population with early maturation status. The treatments affected the hESC-RPE cells so that the ability to respond was lost or the response was weaker compared to the control cells. We have previously shown that the higher maturation level increases the response amplitudes [12], although in this study, the maximum amplitudes were lower at 12 weeks compared to the cells with early maturation status. The comparison of the different maturation levels in Viheriälä et al. [12] was only 4 weeks, and in this study, the comparison is 9 weeks with completely different maturation levels. We also want to highlight that the increased pigmentation at 12 weeks can hinder the detection of the fluorescence signal, thus influencing the observed maximum amplitudes. In addition, the expression of the P2Y_2_ receptor was increased during maturation suggesting that the low maximum amplitudes in control cells at 12 weeks were not due to compromised expression levels of the P2Y_2_. However, the receptor was preserved better after the treatments at the 12 weeks, which could explain the better responses after the treatments at this time point. Differences in Ca^2+^ responses between MG132 and H_2_O_2_ treated cells could be, at least partly, explained by the P2Y_2_ localization (Fig. 5f) which shifts from apical to more cytoplasmic, especially after the MG132 treatment at both time points.

RPE cells are known to be involved in immune responses and thereby secrete several immunomodulatory cytokines under normal conditions of the retina [49]. Their production is tightly regulated and can be modulated via various stimuli such as pathogens or other cytokines [50]. In addition, in some diseases like AMD, secretion of cytokines is dramatically upregulated [50]. RPE transplantation also has a risk of intraocular complications associated with inflammation and elevated levels of cytokines such as IFN-γ which is known to lead to upregulation of HLA-II expression in hPSC-RPE cells and immune reactions in HLA mismatched recipients [51–53]. Of the cytokines studied here, the untreated cells secreted CXCL-12/SDF-1, MIF, Serpin-1/PAI-1, CCL2/MCP-1, and ICAM/CD54, from which the CXCL-12/SDF-1 were secreted only at 3 weeks. In addition, IL6 and IL8 were secreted only after the treatments. Production of some of these cytokines by either primary or hPSC-RPE has been reported also previously by others [53, 54]. Being proinflammatory, these cytokines serve as important initiative signals for protective inflammation against pathogens. However, in the context of cell therapy, their secretion may increase the risk of graft rejection by e.g. chemoattraction of immune cells (CXCL12/SDF-1 [55], CCL2/MCP-1 [56], IL8 [56]) or influence angiogenesis (Serpin-1/PAI-1 [57], CCL2/MCP-1 [56]). The expression of CCL2/MCP-1 was downregulated after the MG132 treatment at both time points as has been shown previously for RPE by Liu et al. [58]. Secretion of MIF has been connected to proliferative vitreoretinopathy [39] and it has been shown to enhance migration and proliferation of RPE cells [59]. Interestingly, in two AMD patients treated with hESC-RPE, spreading of the pigmented area outward of the graft was noticed, potentially indicating migration of the hESC-RPE off the patch [6]. Of note, in our study, secretion of IL6, a mediator both in acute and chronic inflammatory responses, was expressed only after the treatment with H_2_O_2_ at both time points. In addition, IL8 was expressed only after the MG132 treatment at 3-week time point. These findings are consistent with the earlier studies where have been shown the expression of IL6 and IL8 to be upregulated after the stimulation of H_2_O_2_ or MG132 [58, 60]. Despite the reported immunosuppressive properties of RPE cells, the production and induction of several proinflammatory molecules by hESC-RPE suggests that the use of immunosuppression in their transplantation is still essential [51].

RPE cells have robust defense mechanisms against oxidative stress, including pigmentation and efficient antioxidant and degradation systems [3], protecting e.g. tight junctions and therefore preventing the loss of critical RPE functions such as barrier integrity. The findings in the current study suggest that the development or maturation of these protective measures appears to correlate with the maturation of tight junctions and takes several weeks in hESC-RPE, rendering hESC-RPE with early maturation status vulnerable to even sublethal cell stress, potentially impairing critical RPE functions post-transplantation.

## Conclusions

Based on the analyses conducted in this study, the hESC-RPE cells showed improved tolerance to cellular stress at the culture age of 12 weeks in comparison to 3 weeks. Toleration to cellular stress was studied with treatments of chemical stressors MG132 or H_2_O_2_. Treatments affected various hESC-RPE functional properties such as cellular barrier, rate of phagocytosis, Ca^2+^ signaling, and cytokine secretion. From these analyses, TER, phagocytosis, and Ca^2+^ signaling properties were reduced, and CL19 localization was shifted from apical to cytoplasmic at 3-week time point compared to 12 weeks, especially after MG132 treatment. Our results suggest the superiority of the more mature hESC-RPE population for successful cell therapy.

## Supplementary Information

**Additional file 1. Fig. S1:** Ca^2+^ signaling of control, MG132 and H_2_O_2_ treated hESC-RPE cells at time points of 3 and 12 weeks. Number of responding and non-responding cells at timepoints of a) 3 weeks and b) 12 weeks. Both time points include 2–5 replicate measurements. Bar data represents means ± SD. *p < 0.05.

**Additional file 2. Fig. S2:** Replicate of cytokine secretion of hESC-RPE. Intensity levels of secreted cytokines of control cells at a) 3 weeks and b) 12 weeks. Control values are average values from membranes within each experiment (n = 2 for 3 weeks and n = 2 for 12 weeks). Intensity levels after MG132 treatment at c) 3 weeks and b) 12 weeks and after H_2_O_2_ treatment at e) 3 weeks and f) 12 weeks. g)–i) Cytokine array membranes illustrating spot intensities.

**Additional file 3. Fig. S3:** Control membranes illustrating spot intensities from cytokine secretion assays of hESC-RPE at a) 3-week and b) 12-week time points. Exposure time in which the control spots (asterisks) had reached maximal grey value were chosen for each membrane.

## Data Availability

All data used in this study are included in this article and additional files.

## Abbreviations

RPE: Retinal pigment epithelium
hESC-RPE: Human embryonic stem cell-derived retinal pigment epithelium
Ca^2^^+^: Calcium
H_2_O_2_: Hydrogen peroxide
AMD: Age-related macular degeneration
hPSC-RPE: Human pluripotent stem cell-derived retinal pigment epithelium
POS: Photoreceptor outer segment
ATP: Adenosine triphosphate
XF: Xeno-free
KO-DMEM: KnockOut™ Dulbecco’s modified Eagle’s medium
PET: Polyethylene terephthalate
TMRE: Tetramethylrhodamine ethyl ester
FCCP: Carbonyl cyanide 4-(trifluoromethoxy) phenylhydrazone
TER: Transepithelial electrical resistance
RT: Room temperature
PFA: Paraformaldehyde
PBS: Phosphate saline buffer
BSA: Bovine serum albumin
ZO-1: Zonula Occludens
Cx43: Connexin43
CL19: Claudin-19
MIP: Maximum intensity projections
FBS: Fetal bovine serum
Fluo-4 AM: Fluo-4-acetoxymethyl ester
ROI: Region of interest
MIF: Microphage migration factor
CCL2/MCP-1: Monocyte chemoattractant protein-1
ICAM/CD54: Intracellular adhesion molecule 1
CXCL12/SDF-1: Stromal cell-derived factor 1
Serpin-1/PAI-1: Plasminogen activator inhibitor-1
IL8: Interleukin8
IL6: Interleukin6
ROS: Reactive oxygen species
MERTK: MER tyrosine kinase
