# Supplementary figures and images for: Cell maturation influences the ability of hESC-RPE to tolerate cellular stress
Source: Stem Cell Res Ther. 2022 Jan 24;13:30. doi: 10.1186/s13287-022-02712-7 (PMC8785579; doi:10.1186/s13287-022-02712-7)

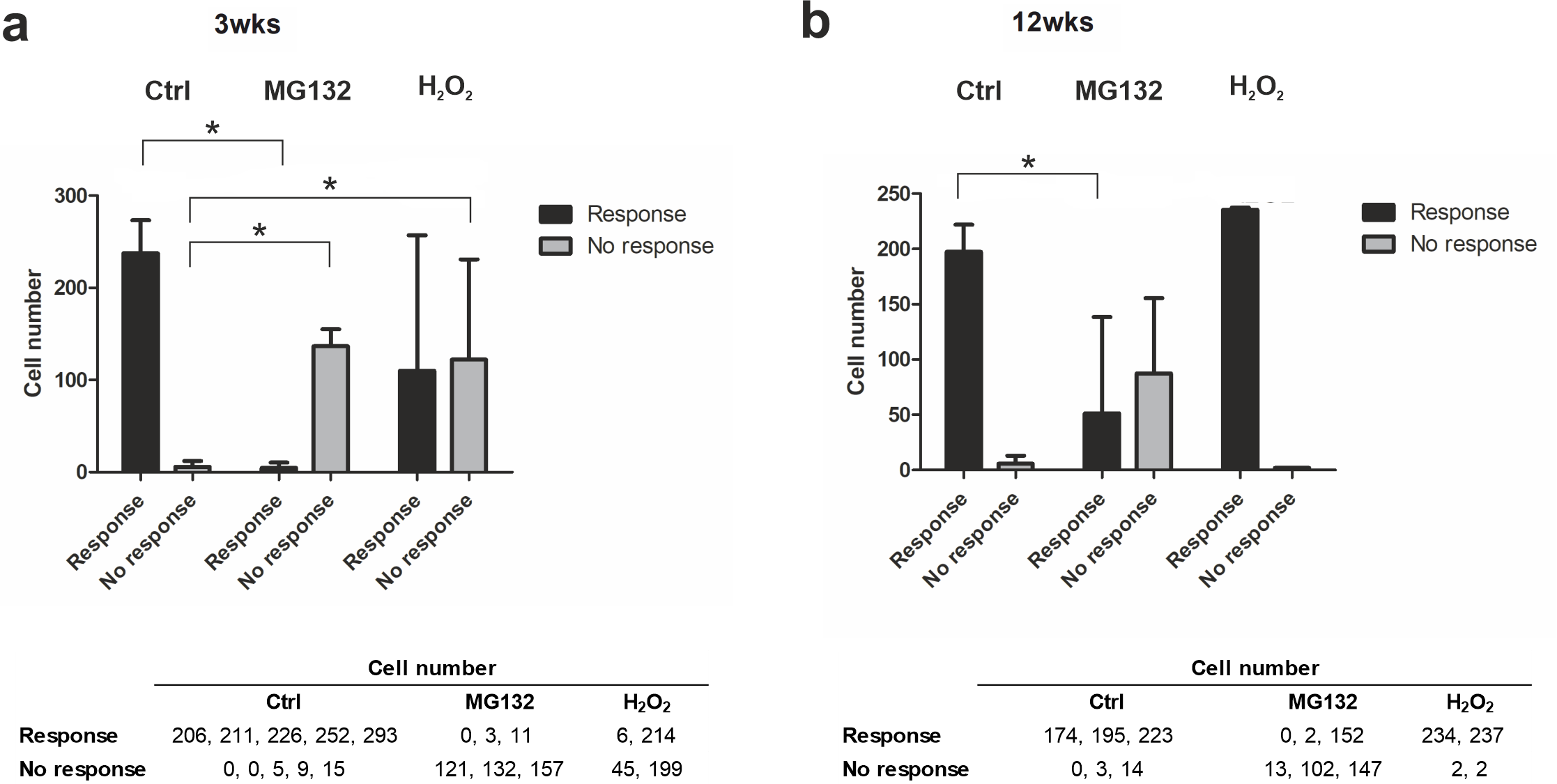

Supplement: Supplementary file 1 — Additional file 1. Fig. S1: Ca2+ signaling of control, MG132 and H2O2 treated hESC-RPE cells at time points of 3 and 12 weeks. Number of responding and non-responding cells at timepoints of a) 3 weeks and b) 12 weeks. Both time points include 2–5 replicate measurements. Bar data represents means ± SD. *p < 0.05. [file 13287_2022_2712_MOESM1_ESM.tif]
